# Bioinformatics analysis of genes related to uveitis and differential gene expression in a model of experimental autoimmune uveitis

**DOI:** 10.1371/journal.pone.0358218

**Published:** 2026-09-21

**Authors:** Kun He, Bingxin Pan, Pengfei Tian, Suyun Yang

**Affiliations:** Quzhou Hospital of Zhejiang Medical and Health Group (Zhejiang Quhua Hospital), Quzhou, Zhejiang, China; University of Texas at El Paso, UNITED STATES OF AMERICA

## Abstract

Uveitis is a sight-threatening disease in which autoimmune and inflammatory reactions play a central role. This study aimed to identify key genes involved in the pathogenesis of uveitis using bioinformatics approaches and to validate their expression in a rat model of experimental autoimmune uveitis (EAU). The GSE66936 microarray dataset, comprising transcriptomes of CD14 and CD16 monocytes from five patients with uveitis and four healthy donors, was downloaded from the Gene Expression Omnibus, and known uveitis-related targets were retrieved from six disease-gene databases. Differentially expressed genes (DEGs) were identified and subjected to Gene Ontology (GO) and Kyoto Encyclopedia of Genes and Genomes (KEGG) enrichment analyses. A protein-protein interaction network was constructed using STRING, and hub genes were screened with Cytoscape and evaluated by receiver operating characteristic (ROC) curve analysis. EAU was induced in Sprague-Dawley rats by immunization with interphotoreceptor retinoid-binding protein (IRBP), and ocular inflammation was assessed by slit-lamp examination and histopathology. The expression of the hub genes was validated in ocular, liver, and spleen tissues by reverse transcription-quantitative polymerase chain reaction and enzyme-linked immunosorbent assay. A total of 139 uveitis-related genes were identified; these were enriched in inflammatory response, immune response, viral defense response, and the TNF and NF-κB signaling pathways. Ten hub genes (*IL17A, IL18, TLR2, IL2, TLR4, IL10, IL1B, IL6, TNF*, and *CCL2*) were identified, each with an area under the ROC curve greater than 0.6. The EAU model was successfully established, with significantly greater anterior segment inflammation than in controls, and the mRNA and protein levels of the hub genes were significantly elevated in the ocular and peripheral tissues of EAU rats (p < 0.05). These findings reveal key genes and pathways involved in the pathogenesis of uveitis and provide candidate targets for its diagnosis and treatment.

## Introduction

Uveitis is a complex disease that is influenced by genes and the environment, with an unknown etiology and a complex pathogenesis; the main factors responsible are autoimmune and inflammatory reactions. This condition occurs mostly in young adults and often recurs; in addition, uveitis is often combined with systemic autoimmune diseases, cannot be prevented and is difficult to treat [1]. Uveitis is the fourth leading cause of acquired blindness [2–4] and affects approximately 1 in 1000 people [5]. With the advancement of science and technology, the application of microarray technology and bioinformatics analysis has provided new methods and perspectives to reveal the genetic background and functional mechanism of uveitis.

The aim of this study was to identify key genes associated with uveitis by bioinformatics methods and to explore the roles of these genes in biological functions and signaling pathways by Gene Ontology (GO) and Kyoto Encyclopedia of Genes and Genomes (KEGG) analyses. We aimed to identify the molecular mechanisms associated with the pathogenesis of uveitis by constructing a protein-protein interaction (PPI) network and analyzing differentially expressed key genes using correlation plug-ins. In addition, we further investigated the potential molecular mechanisms of uveitis by inducing an experimental rat model of autoimmune uveitis (EAU) and validating changes in differentially expressed genes (DEGs). These findings provide new insights and approaches for a deeper understanding of the pathogenesis of uveitis and the development of targeted therapeutic strategies. Through the integrated use of bioinformatics analysis and animal experimental validation, this study not only reveals the bioinformatics characteristics of uveitis-related genes, but also provides new perspectives and evidence for understanding its complex pathogenesis, thus providing a scientific basis for the development of new therapeutic strategies.

It is important to note that the GSE66936 dataset characterizes transcriptomes from human CD14 and CD16 monocytes (innate immunity), whereas the EAU model evaluates CD4 + T cell-mediated adaptive immunity. We selected this dual-strategy approach because monocytes and macrophages serve as critical antigen-presenting cells (APCs) that initiate and orchestrate adaptive T cell responses in uveitis [6]. Monocyte-derived cytokines (IL-6, IL-1β, TNF-α) and chemokines (CCL2, CCL5) provide essential signals that drive Th1 and Th17 cell polarization [7], bridging the innate-adaptive immune interface in autoimmune uveitis.

## Materials and methods

### Data sources

The Gene Expression Omnibus (GEO) (http://www.ncbi.nlm.nih.gov/geo) database [8] is an open-access resource for functional genomic data, including gene expression data and microarrays. We downloaded a specific uveitis gene expression dataset from the GEO that had been specifically acquired by the Affymetrix GPL 570 platform and the Affymetrix Human Genome U133 Plus 2.0 Array (GSE66936).The annotations provided by the platform contained the information required to convert probes into their respective gene symbols. The GSE66936 dataset provides data acquired from CD14 and CD16 monocytes harvested from five individuals diagnosed with uveitis and four healthy donors. By applying “uveitis” as the primary keyword, we searched a series of six databases for established disease targets. These databases represent acknowledged databases related to disease-related genes: the TTD (http://database.idrb.cqu.edu.cn/TTD/), the DrugBank database (https://www.drugbank.ca/), the OMIM database (https://www.omim.org/), the GAD database (http://geneticassociationdb.nih.gov/), the PharmGKB database (https://www.pharmgkb.org/index.jsp), and the DisGeNET database (https://www.disgenet.org/). Following the elimination of duplicate data, we compiled a list of known disease targets for uveitis.

### Screening for differentially expressed genes

The GEO2R tool (http://www.ncbi.nlm.nih.gov/geo) was used to compare gene expression between monocytes from patients with uveitis and those from healthy donors.As an interactive web tool, GEO2R allows users to compare two or more groups of samples within a GEO series to identify differentially expressed genes (DEGs) under experimental conditions [9]. We applied adjusted P-values and Benjamini and Hochberg false discovery rates (FDRs) to achieve a balance between uncovering statistically significant genes and constraining the emergence of false positives.Probe sets without a corresponding gene symbol were removed, and probe sets mapping to the same gene symbol were averaged.

### PPI network construction

Next, the online STRING (http://string-db.org) (version 10.0) database [10] was used to predict a protein-protein interaction (PPI) network. Analyzing the functional interactions between proteins can provide valuable insights into the mechanisms underlying disease onset and progression. In the present study,interactions with a combined score greater than 0.4 were considered statistically significantand visualized using Cytoscape software, and thenthe key genes were predicted and defined as hub genes using the cytoHubba plug-in of Cytoscape. Additionally,the identified hub genes were subjected to ROC curve analysis for validation. The ROC analysis was performed using SPSS Statistics software(version 25.0; IBM Corp., Armonk, NY, USA), allowing for the accurate evaluation of gene significance in uveitis pathogenesis.

### Gene ontology and KEGG enrichment analysis

Following the identification of DEGs, we performed enrichment analysis using the KEGG and GO databases. These well-established bioinformatics resources were accessed via the biological annotation tool, DAVID (Database for Annotation, Visualization, and Integrated Discovery, Version 6.7) [11]. As an online resource, DAVID amalgamates a variety of gene and protein functional annotation information, enabling researchers to extract valuable biological information. The KEGG database was utilized to decipher higher-level functions and biological systems from the large-scale molecular datasets produced by high-throughput experimental techniques [12]. The GO database was employed to annotate genes and analyze the biological processes associated with these genes [13]. DEGs were subjected to biological analysis using the DAVID tool, with a significance threshold set at p < 0.05.

### Animal experiments

#### Animals and ethics statement.

In total, 20 healthy male Sprague-Dawley (SD) rats (4–6 weeks of age, weight: 180–200 g) were used in this study. Before the start of the experiment, the eyes of the 20 rats were routinely examined to exclude ocular and systemic diseases (any rats affected by these diseases were excluded from the experiment). All remaining rats were fed for 1 week to adapt to their environment.The laboratory was specific-pathogen-free (SPF) classifiedand the environment was maintained at a room temperature of 24 ± 2°C, a relative humidity of 50–60%, with good ventilation. All rats were fed with regular chow and drinking water.This study was carried out in strict accordance with the recommendations in the Guide for the Care and Use of Laboratory Animals and the ARVO Statement for the Use of Animals in Ophthalmic and Vision Research. The protocol was approved by the Experimental Animal Ethics Committee of Quzhou Hospital of Zhejiang Medical and Healthcare Group (Protocol Number: 2023HCO1LS012). All surgical procedures were performed under sodium pentobarbital anesthesia, and all efforts were made to minimize animal suffering.

#### Main reagents and instruments.

Interphotoreceptor retinoid-binding protein (IRBP) was purchased from Shanghai Bioengineering Co., Ltd. (batch no. P21622). *Mycobacterium tuberculosis* H37Ra was purchased from BD Difco (USA; batch no. M4537). Freund’s complete adjuvant (CFA) was purchased from Sigma (USA; batch no. F5881). Eyeball fixative was purchased from Wuhan Google Biotechnology Co., Ltd. Hematoxylin-eosin (HE) staining solution was purchased from Jiangsu Kaiji Biotechnology Co., Ltd. (lot no. 20160801). Phosphate-buffered saline (PBS, dry powder) was purchased from Biosharp (batch no. 10100147). RNAiso Plus, the PrimeScript RT Reagent Kit with gDNA Eraser (Perfect Real Time), primers, and SYBR Premix Ex Taq II were purchased from TaKaRa (Dalian, Liaoning, China). ELISA kits were purchased from eBioscience (USA). The instruments used in this study included a StepOnePlus Real-Time PCR System (Applied Biosystems, USA), a 5427R low-temperature centrifuge (Eppendorf, Germany), an HM355S automatic paraffin microtome (Thermo Scientific, USA), and a YZ5X slit-lamp microscope (66 Vision-Tech, Suzhou, China).

#### Establishment and grouping of the EAU rat model.

After the exclusion of rats with systemic and ocular abnormalities, and acclimatization, the remaining animals were sequentially numbered and randomly divided into a normal control (Control, CTRL) group and an EAU model group by the random number table method, with 10 rats in each group. The EAU model was established by the subcutaneous injection of IRBP, as reported previously [14]. PBS containing IRBP was prepared on an ultra-clean table and mixed with 10 mg of *Mycobacterium tuberculosis* H37RA powder, 1.4 mL of PBS and 2 mL of CFA to obtain 4 mL of emulsion which was injected subcutaneously into the middle of the foot pads of the rats. The needle was advanced upwards to the upper end of the caudal vertebra before we slowly injected 0.6 mL of the mixed emulsion into the upper end of the caudal vertebra. The CTRL group was injected with an equal dose of saline at the same site. Inflammatory reactions in the anterior segment of the eye were observed with a slit lamp, and after 12 days of slit lamp observation, the rats were euthanized. From each rat, one eyeball was removed for histopathological observation; the other eyeball was frozen in liquid nitrogen and ground into a dry powder for real-time PCR detection.

#### Slit-lamp observation of inflammatory reactions.

Inflammatory reactions in the anterior segment of each eye were observed and photographed under a slit-lamp microscope each dayafter modeling, for a total of 12 days. The degree of inflammation in the anterior segment of the eye was evaluated according to Caspi’s scoring criteria.

#### HE staining and histopathological observation of eyeball tissue.

Eight rats were randomly selected from the two groups on days 4, 8 and 12, anesthetized by an intraperitoneal injection of sodium pentobarbital (40 mg/kg), and then euthanized by cervical dislocation under deep anesthesia. The skin surrounding the eyes wasdisinfected with Anerdian type III antiseptic (iodophor), and the eyeballs were separated from the peripheral tissues, including the lid conjunctiva. Next, we cut the optic nerves and blood vessels of the distal eyeballs withsterile ophthalmic microsurgical instruments, and then completely removed the eyeballs bilaterally. The eyes were fixed in 4% paraformaldehyde solution overnight and paraffin blocks were prepared for sectioning and HE staining. Sagittal sections (4 µm in thickness) were cut through the pupillary optic papillary axis. Tissue sections were then stained with HE and observed by microscopy (Leica DM4000). Three fields of view were selected from each section near the cornea, the anterior chamber ciliary processes and the retina, and pathology was graded according to Caspi’s histopathological scoring criteria for the eye. Photographs and counts were acquired and analyzed by two experienced ophthalmology researchers.

#### RT-qPCR detection of inflammatory factor mRNA expression.

Total RNA was extracted from ocular tissues that had been snap-frozen and ground in liquid nitrogen using RNAiso Plus (the TRIzol method). The concentration of each RNA sample was determined using a NanoDrop 2000 spectrophotometer (Thermo Fisher Scientific, USA), and mRNA was reverse transcribed into cDNA using the PrimeScript RT Reagent Kit with gDNA Eraser (TaKaRa, Dalian, China) according to the manufacturer’s instructions. Quantitative real-time PCR was then performed using SYBR Premix Ex Taq II on a StepOnePlus Real-Time PCR System (Applied Biosystems, USA) in a total reaction volume of 15 μL (7.5 μL of SYBR Premix Ex Taq II, 2.0 μL of cDNA, 1.5 μL of each gene primer, and 4.0 μL of ddH2O). The reaction conditions were pre-denaturation at 95°C for 10 min, followed by 40 cycles of denaturation at 95°C for 15 s, annealing at 60°C for 15 s, and extension at 72°C for 15 s. A melting curve was generated at 95°C for 10 s and 60°C for 60 s, and all reactions were performed in triplicate. Gene expression levels were calculated using the 2^-ΔΔCt^ method and normalized to *β-actin*. The primer sequences are shown in Table 1.

**Table 1:** Primer sequences used for RT-qPCR.

Gene	Forward primer sequence (5′-3′)	Reverse primer sequence (5′-3′)
*β-actin*	TGCTATGTTGCCCTAGACTTCG	GTTGGCATAGAGGTCTTTACGG
*IL17A*	TGAGCTGCAGAGAGTCAAGAATAG	CATGCAACACAGGGGACTAATTC
*IL18*	GCAGTAATACGGGAGCATAAA	ATCCTTCACAGATAGGGTCA
*TLR2*	CTGAGATCTAGAGCTATAGCTA	ATAGCGATATGCGATATGCGATA
*IL2*	GCAGCGTGTGTTGGGATTTGAC	TGCTGGCTCATCATCGAATTG
*TLR4*	TCATCCAGGAAGGCTTCCAC	GGCGATACAATTCGACCTGC
*IL10*	CAGACCCACATGCTCCGAGA	CAAGGCTTGGCAACCCAAGTA
*IL1B*	GCCAACAAGTGGTATTCTCCA	TGCCGTCTTTCATCACACAG
*IL6*	RGGAGTTCCGTTTCTACCTGG	GCCGAGTAGACCTCATAGTG
*TNF*	CCACCACGCTCTTCTGTC	GCTACGGGCTTGTCACTC
*CCL2*	GATCTGTGCTGACCCCAATAAGG	GGTGCTGAAGTCCTTAGGGTTGA

##### ELISA detection of DEG protein expression in tissues.

First, we isolated liver and spleen tissues from the CTRL group and the EAU group. These were quickly immersed in liquid nitrogen. Then, we added 400 μL of lysis buffer (2 μL of protease inhibitor for every 1 mL of RIPA lysis buffer) and the tissues were ground with a glass homogenizer. Subsequently, the lysates were sonicated for 20 minutes using an ultrasonic cell disruptor. After sonication, the lysate was centrifuged at 8000 rpm at 4°C for 20 minutes. The supernatant was then transferred to a new microcentrifuge tube to allow protein concentration to be determined. Finally, ELISA kits were used to determine the protein concentrations of IL-17A, IL-18, TLR2, IL-2, TLR4, IL-10, IL-1β, IL-6, TNF, and CCL2 in liver and spleen tissues from experimental animals.

### Statistical analysis

Experimental data were analyzed using SPSS version 20.0 (IBM Corp., Armonk, NY, USA), and RT-qPCR data were analyzed using GraphPad Prism version 9.5.1 (GraphPad Software, San Diego, CA, USA). Data are expressed as the mean ± standard deviation (SD). All data were tested for normality and homogeneity of variance. Comparisons between two groups were performed using the independent-samples t-test when the data were normally distributed with homogeneous variance; otherwise, non-parametric tests (the Mann-Whitney U test) were used. Comparisons among multiple groups were performed by one-way analysis of variance (ANOVA) followed by the least significant difference (LSD) post hoc test. A two-tailed p < 0.05 was considered statistically significant (*p < 0.05, **p < 0.01, ***p < 0.001, ****p < 0.0001).

## Results

### Screening for genes involved in the pathogenesis of uveitis

**Fig 1:**
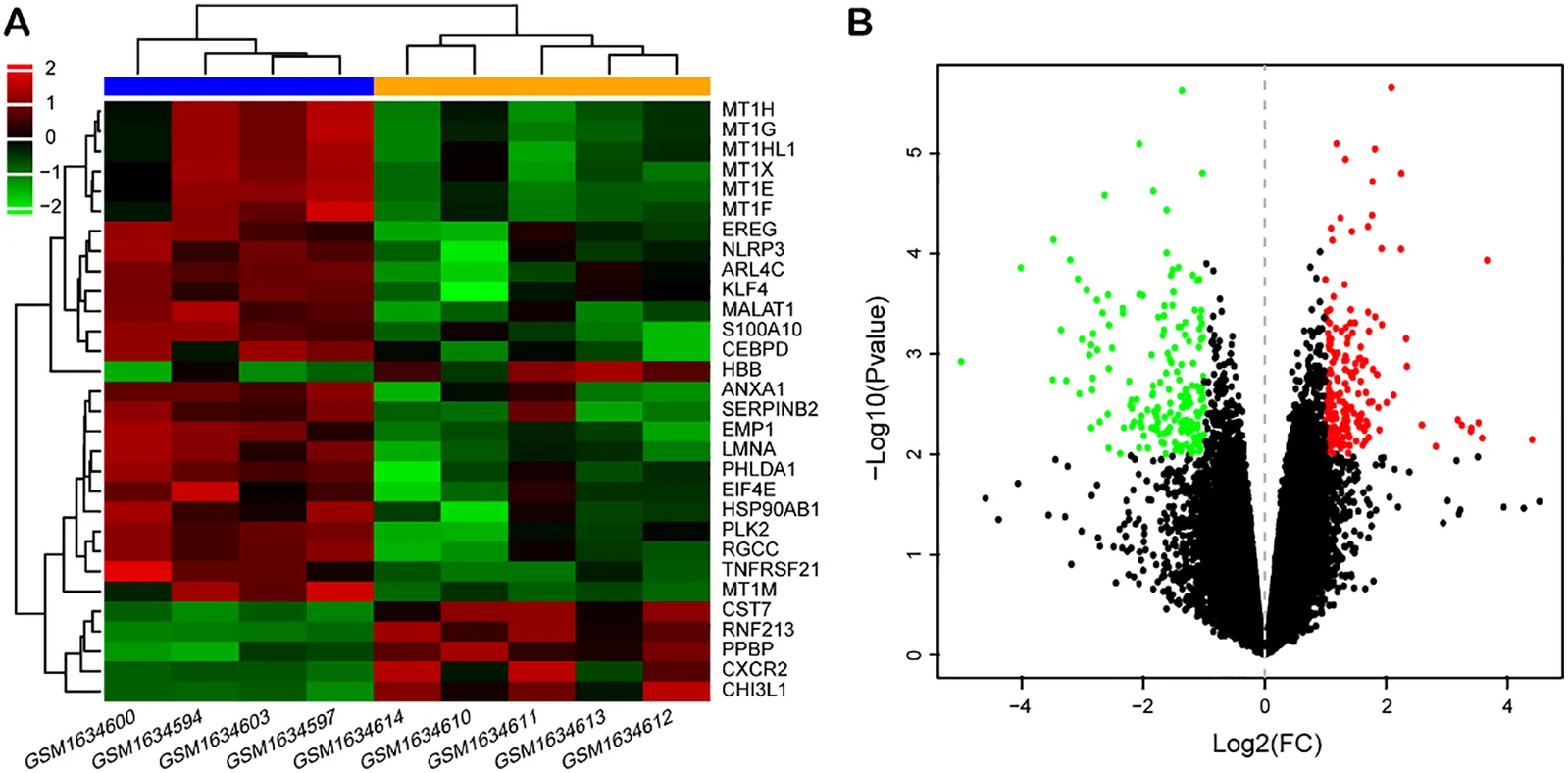
Distribution of DEGs in the GSE66936 dataset. (A) Cluster heatmap; (B) volcano plot.

Potential targets were predicted using the GEO database (http://www.ncbi.nlm.nih.gov/geo). The top 30 DEGs identified from the GSE66936 dataset are shown in the clustering heatmap.The distribution of DEGs is shown in the form of cluster and volcano plots (Fig 1A and 1B)

**Fig 2:**
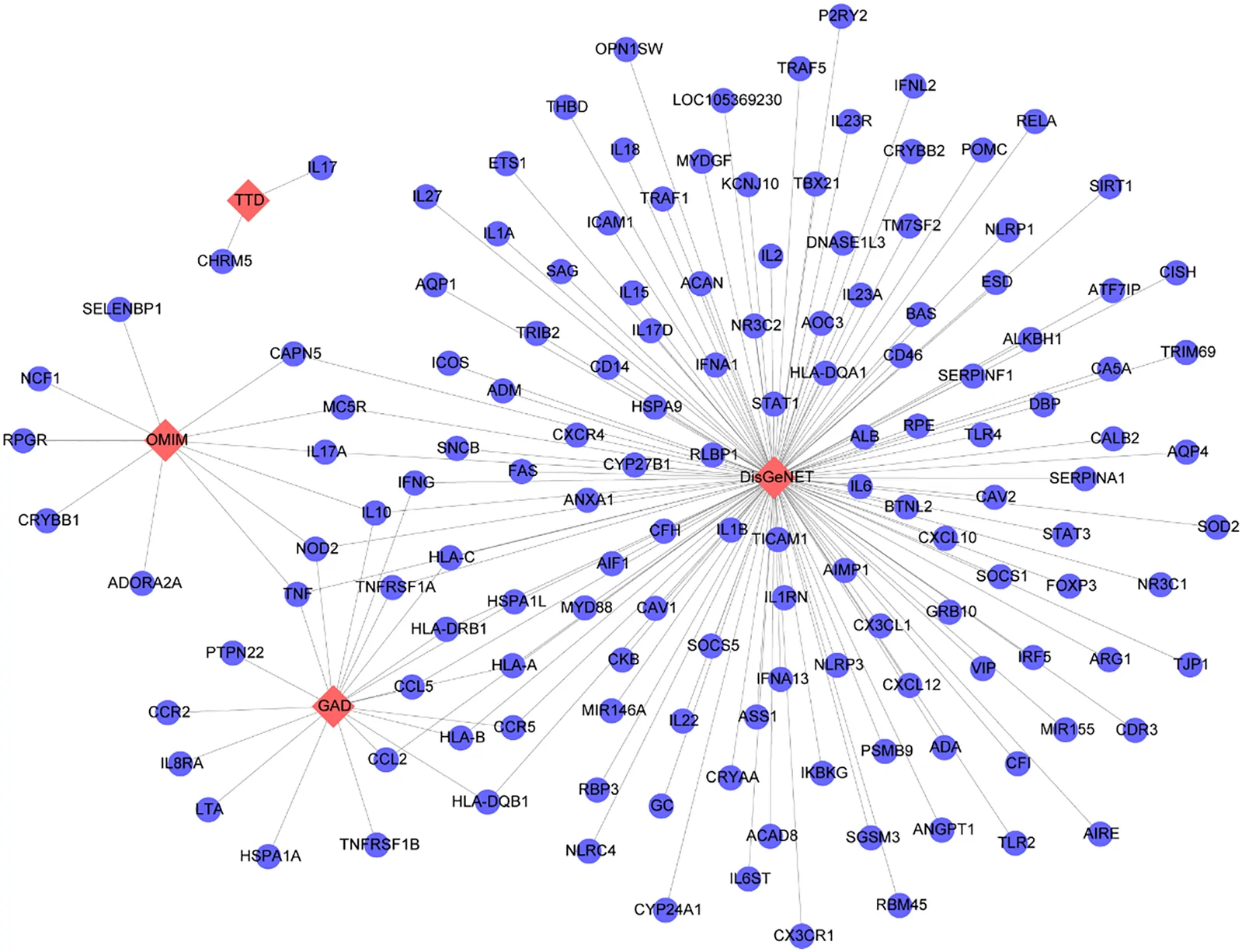
Uveitis-related target genes identified by database searches. Known uveitis-related targets retrieved from six internationally recognized disease-gene databases.

Known uveitis-related target genes were also identified by searching disease-related target databases. In this paper, six internationally recognized disease gene-related databases were searched, and a large number of target genes closely related to the occurrence and development of uveitis were acquired: 23, 2, 11, 0, 0 and 129 targets were identified in the GAD, TTD, OMIM, DrugBank,PharmGKBand DisGeNET databases, respectively, and a total of 139 known targets related to uveitis wereidentified by removing duplicates (Fig 2).

### PPI network, hub genes, and validation results

**Fig 3:**
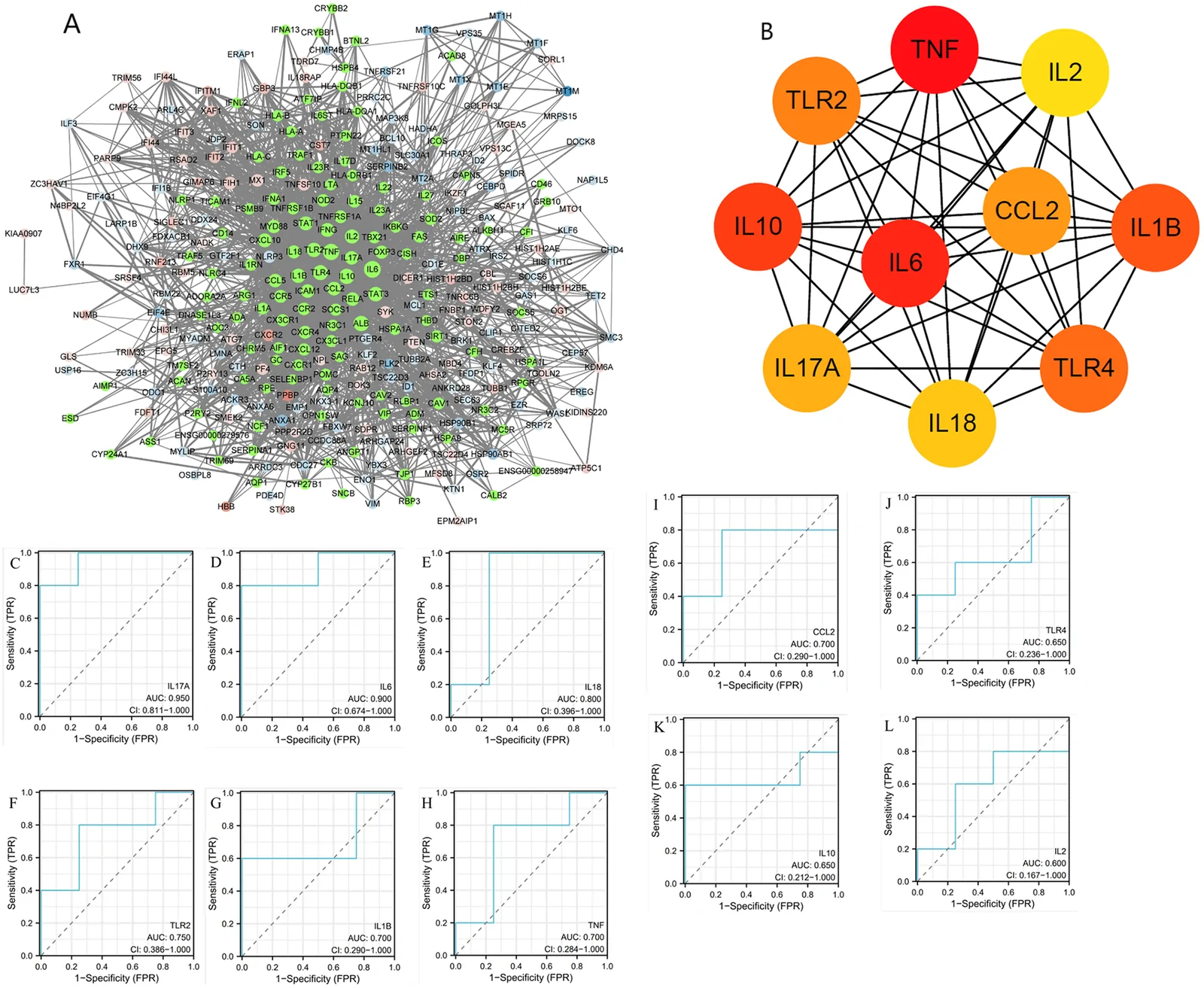
PPI network construction and hub gene screening. (A) PPI network of the DEGs; nodes represent proteins, and edges represent interactions between proteins. (B) The ten hub genes identified from the PPI network using the cytoHubba plug-in of Cytoscape; the color change from red to yellow indicates scores from high to low. (C-L) Receiver operating characteristic (ROC) curves of the ten hub genes and their areas under the curve (AUCs).

A PPI network of the differentially expressed proteins was constructedusing the STRING tool;the network comprised 311 nodes and 2632 interactions (Fig 3A). The nodes with the highest connectivity, referred to as key genes, were filtered using the cytoHubba plug-in of Cytoscape, and we identified the top ten key genes, *IL17A, IL18, TLR2, IL2, TLR4, IL10, IL1B, IL6, TNF* and *CCL2* (Fig 3B). The accuracy of the characterized genes was assessed by ROC curves; these curves were constructed for each gene to assess diagnostic efficacy, and the corresponding area under the curve (AUC) was calculated. Analysis showed thatthe AUC for each gene was > 0.6 (Fig 3C-3L). The hub genes play an important role in the process of the autoimmune and inflammatory responses in uveitis.

### GO and KEGG enrichment analysis of differentially expressed genes

**Fig 4:**
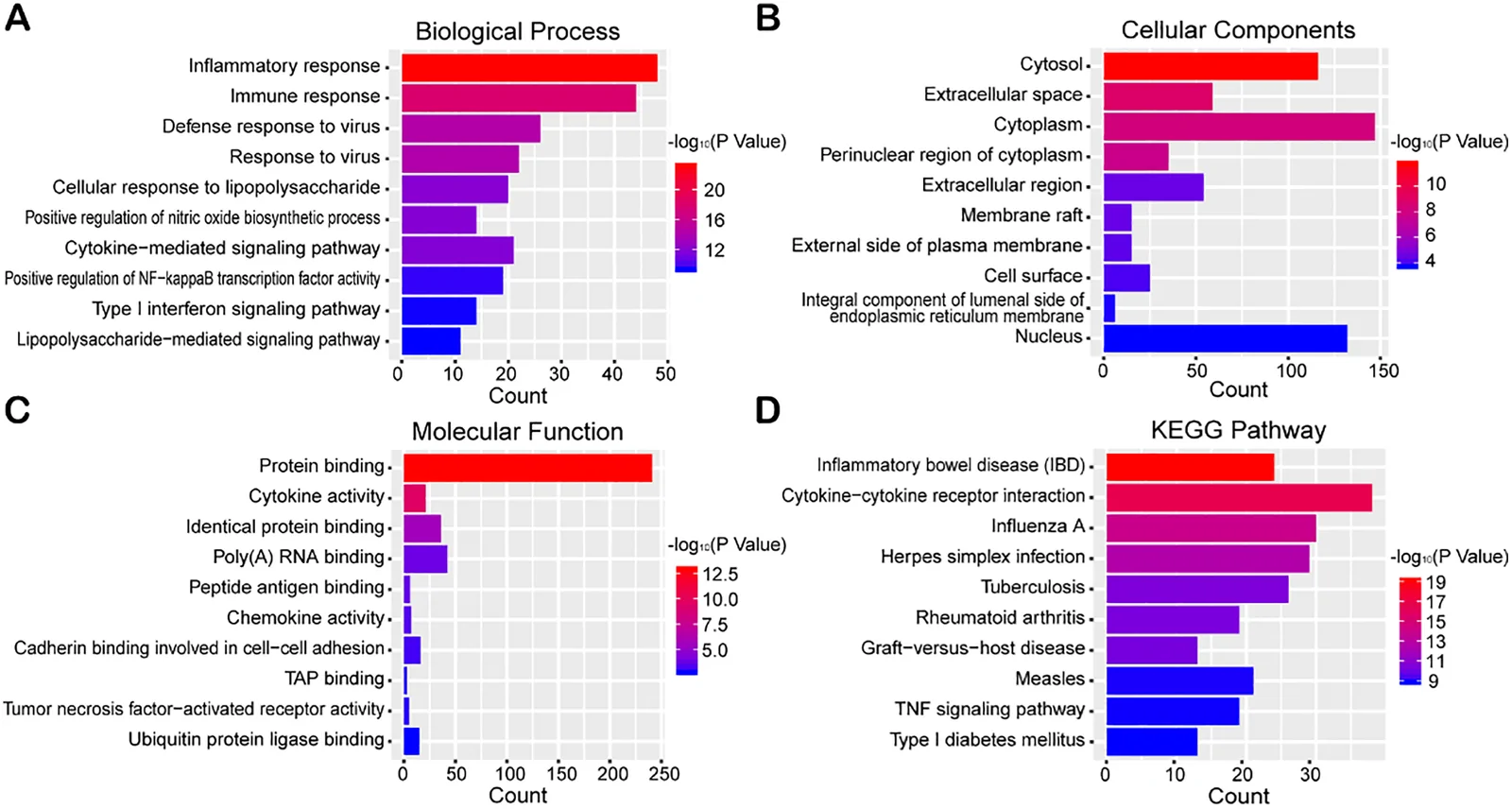
GO and KEGG enrichment analysis of uveitis-related genes. (A) Biological process; (B) cellular component; (C) molecular function; (D) KEGG signaling pathways.

To analyze the biological classification of the DEGs, we performed GO and KEGG enrichment analyses using the DAVID tool. GO analysis showed that the biological processes enriched in the DEGs included inflammatory response, immune response, defense response to virus, and cellular response to lipopolysaccharide (Fig 4A). The enriched cellular components were mainly the cytosol, cytoplasm, and nucleus (Fig 4B). The enriched molecular functions included protein binding, cytokine activity, protein homodimerization activity, and cadherin binding involved in cell-cell adhesion (Fig 4C).

The results of the KEGG analysis are shown in Fig 4D and Table 2. **The signaling pathways associated with targets of uveitis were mainly enriched in cytokine-cytokine receptor interaction, including the TNF signaling pathway, NF-κB signaling pathway, NOD-like receptor signaling pathway, Toll-like receptor signaling pathway, Jak-STAT signaling pathway, and the Cytosolic DNA-sensing pathway.**

**Table 2:** KEGG enrichment analysis of uveitis-related genes.

KEGG pathway	p-value	Enriched genes
Cytokine-cytokine receptor interaction	2.55E-17	TNFRSF21, CCL2, TNF, IL6ST, IL18, CXCR2, PF4, IL15, CX3CL1, CCL5, CXCL12, IL10, CXCL10, TNFRSF1A, TNFRSF1B, IFNA1, IL17A, IFNL2, IL23A, CXCR4, IFNG, IL1B, FAS, LTA, IL1A, IL6, IL23R, IL18RAP, ACKR3, IL22, TNFRSF10C, TNFSF10, PPBP, CCR5, CCR2, CX3CR1, IFNA13, IL2
TNF signaling pathway	1.33E-09	TRAF1, ICAM1, IL6, TNF, CCL2, RELA, CX3CL1, IL15, CCL5, CXCL10, TNFRSF1A, TNFRSF1B, NOD2, IKBKG, MAP3K8, IL1B, FAS, TRAF5, LTA
NF-κB signaling pathway	2.37E-08	TRAF1, ICAM1, BCL10, TNF, RELA, TLR4, CXCL12, TNFRSF1A, MYD88, IKBKG, TICAM1, IL1B, TRAF5, CD14, LTA, SYK
NOD-like receptor signaling pathway	5.04E-08	HSP90AB1, IL6, TNF, CCL2, RELA, IL18, NLRP3, CCL5, NLRP1, NOD2, NLRC4, IKBKG, IL1B
Toll-like receptor signaling pathway	5.66E-08	IL6, TNF, RELA, TLR2, TLR4, STAT1, CCL5, CXCL10, IFNA1, MYD88, IRF5, TICAM1, IKBKG, MAP3K8, IL1B, IFNA13, CD14
Jak-STAT signaling pathway	4.34E-06	IL6, IL23R, IL6ST, SOCS1, SOCS5, IL15, STAT1, IL22, IL10, CISH, STAT3, IFNA1, IFNL2, IL23A, IFNG, IFNA13, IL2
Chemokine signaling pathway	7.10E-06	CCL2, NCF1, RELA, CXCR2, GNG11, PF4, CX3CL1, STAT1, CCL5, CXCL12, STAT3, CXCL10, PPBP, CCR5, CXCR4, CCR2, CX3CR1, IKBKG, WASL
Cytosolic DNA-sensing pathway	5.02E-04	IL6, IFNA1, IL18, RELA, IKBKG, IL1B, IFNA13, CCL5, CXCL10

### Experimental validation

#### Inflammatory manifestations and clinical scores of the anterior segment in EAU rats.

**Fig 5:**
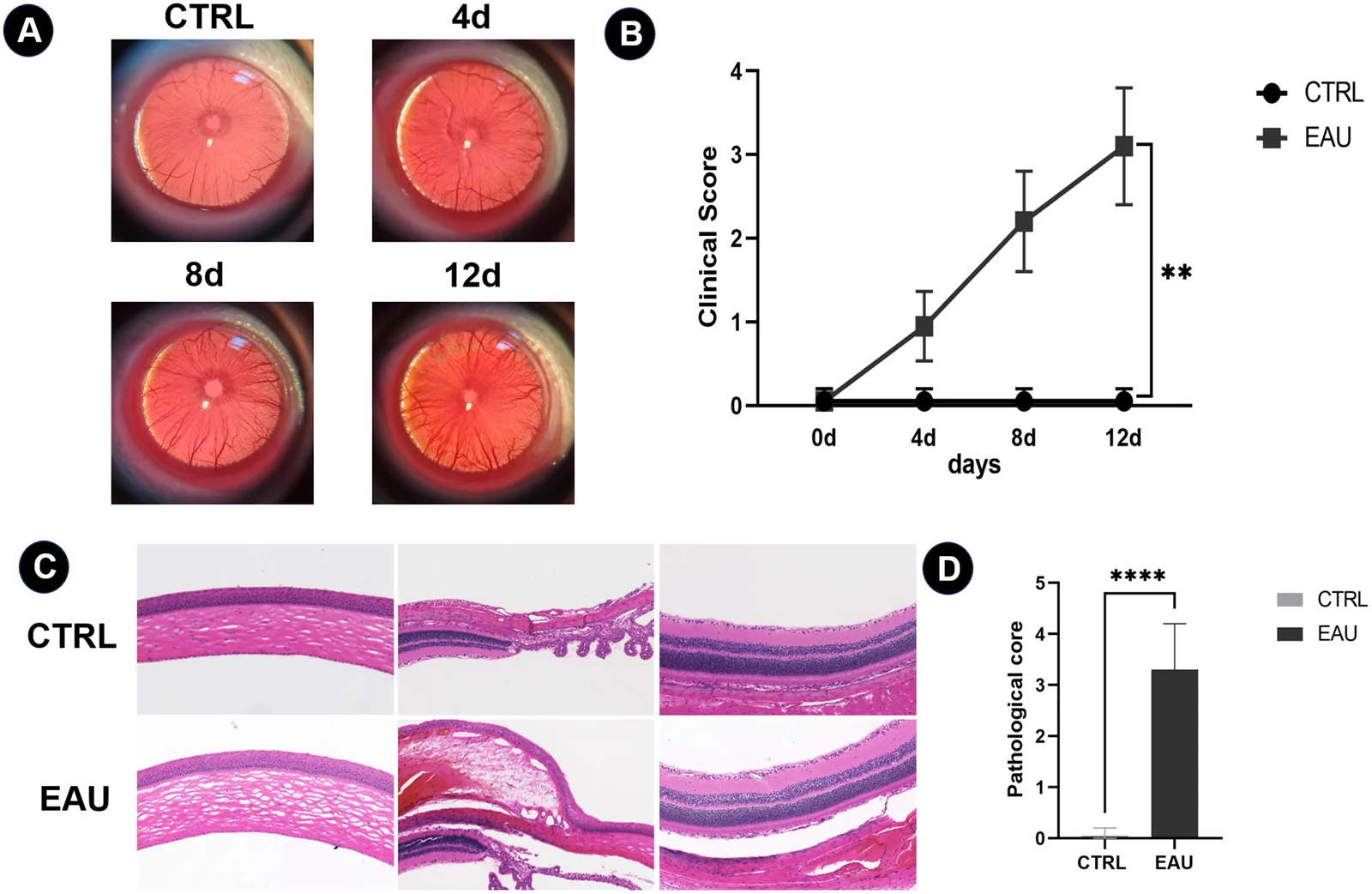
Assessment of IRBP-induced ocular inflammation in the EAU rat model. (A) Representative slit-lamp photographs of the anterior segment of the eye in control rats and in EAU rats at different time points after immunization. (B) Clinical scores of control and EAU rats at different time points. (C) Representative images of ocular histopathology (HE staining) in control rats and in EAU rats 12 days after immunization. (D) Histopathological scores of control and EAU rats 12 days after immunization. n = 10; **p < 0.01, ***p < 0.001.

Normal control rats had no congestion and edema of the iris blood vessels, no congestion of the corneal limbal blood vessels, a clear anterior chamber, no inflammatory reaction, and a clinical score of zero. Mild dilatation of the iris blood vessels and congestion of the corneal limbal blood vessels were seen under the slit-lamp microscope in the EAU group on the 4th day post-immunization, with a clinical score of 0.95 ± 0.41. On days 8 in the EAU group, increased dilatation and congestion of the iris blood vessels,clouding of the anterior chamber, keratic precipitates (KP), aqueous flare, and miosis, with a clinical score of (2.2 ± 0.6). On days 11–14, we observed markedly dilated and congested iris blood vessels,turbid aqueous humor, a shallow anterior chamber, hypopyon, KP, greyish-white flocculent exudates on the pupillary margin, and pupillary membrane occlusion, Inflammation was most severe on day 12, with a clinical score of 3.1 ± 0.7. The clinical scores of the EAU group were significantly higher than those of the control group from day 4 post-immunization onward (p < 0.05) (Fig 5A).The inflammation score curves of the normal control and model groups (Fig 5B) showedthat the EAU model had been successfully established.

#### Histopathological examination of the eyes of EAU rats.

HE staining results showed that in the EAU model group, there was obvious corneal stromaledema, and inflammatory cells were seen in the anterior chamber. The iris and ciliary bodies of rats in the EAU model group were adherent, swollen, and disorganized, and inflammatory cell infiltration was evident. A small amount of inflammatory cell infiltration and localized retinal folds appeared in the retina and vitreous body of the rats in the EAU model group; the structure of the retina was disorganized and edematous, and the mean pathological score was 3.3 ± 0.9. The eyes of rats in the normal control group had a clear structure, the layers were well arranged, and no inflammatory cell infiltration was seen;the pathological score was 0.The histopathological scores of the eyes of rats in the EAU model group were significantly higher than those of the normal control group(p < 0.05) (Fig 5C and 5D).

### RT-qPCR detection of DEG mRNA expression in the ocular tissues of EAU rats.

**Fig 6:**
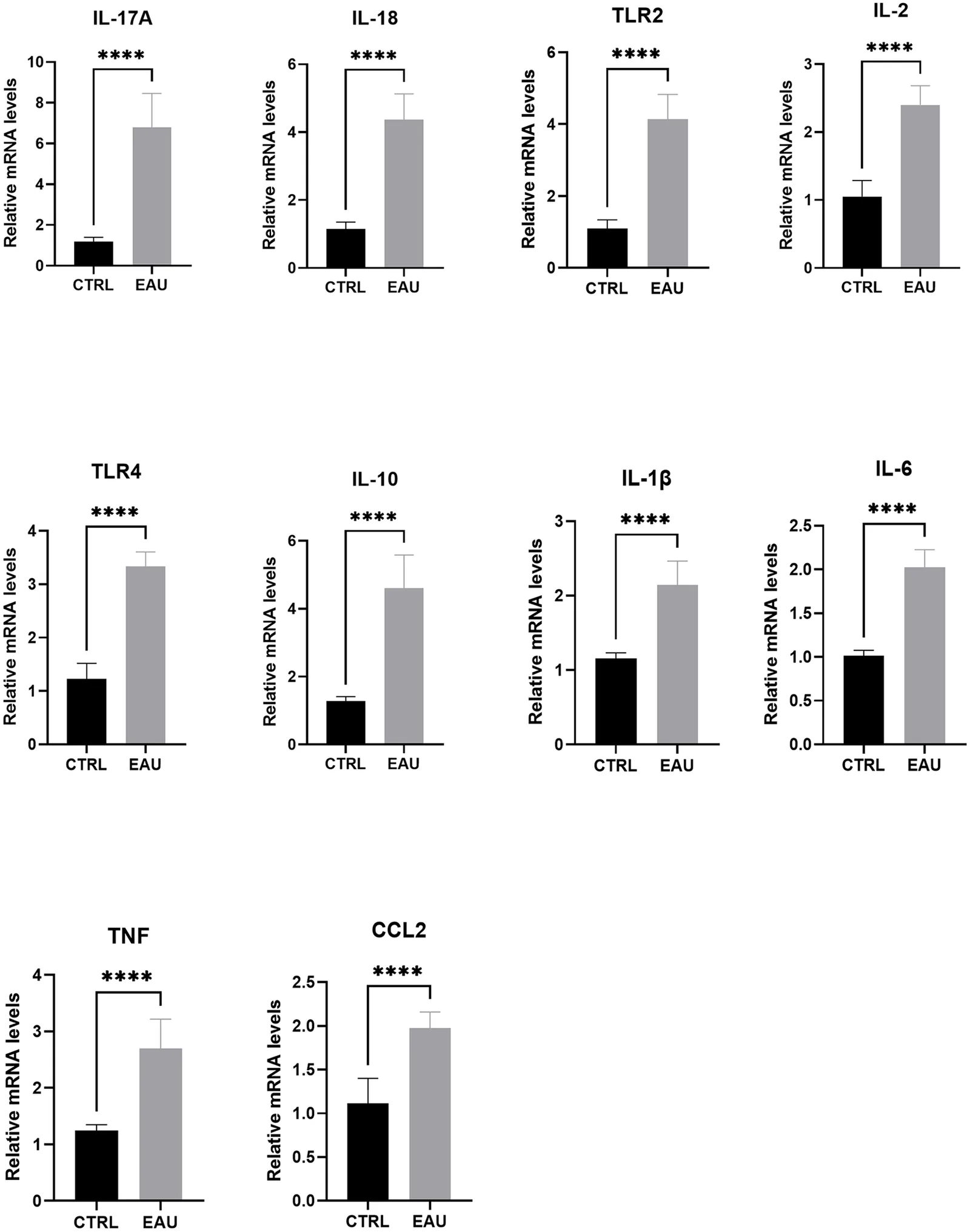
mRNA expression levels of the hub genes in the ocular tissues. mRNA expression levels of *IL17A, IL18, TLR2, IL2, TLR4, IL10, IL1B, IL6, TNF*, and *CCL2* in the ocular tissues of control and EAU rats 12 days after immunization. n = 10; ***p < 0.001.

qRT-PCR analysis showed that the mRNA levels of *IL17A, IL18, TLR2, IL2, TLR4, IL10, IL1B, IL6, TNF* and *CCL2* were significantly elevated in the ocular tissues of EAU rats in the model group as compared with those of the blank control group (p < 0.001) (Fig 6). These results indicated that the DEGs, including inflammation-related key genes, were up-regulated, and that several important pathways were activated, including cytokine-cytokine receptor interaction, the TNF signaling pathway,and the NF-κB signaling pathway. The analysis also indicated that the mRNA expression of DEGs associated with the NF-κB signaling pathway, Nod-like receptor signaling pathway, and Toll-like receptor signaling pathway was statistically significant (P < 0.001). These differentially expressed genes (DEGs) and pathways may play an important role in the exacerbation of inflammation caused by the onset of uveitis.

**Fig 7:**
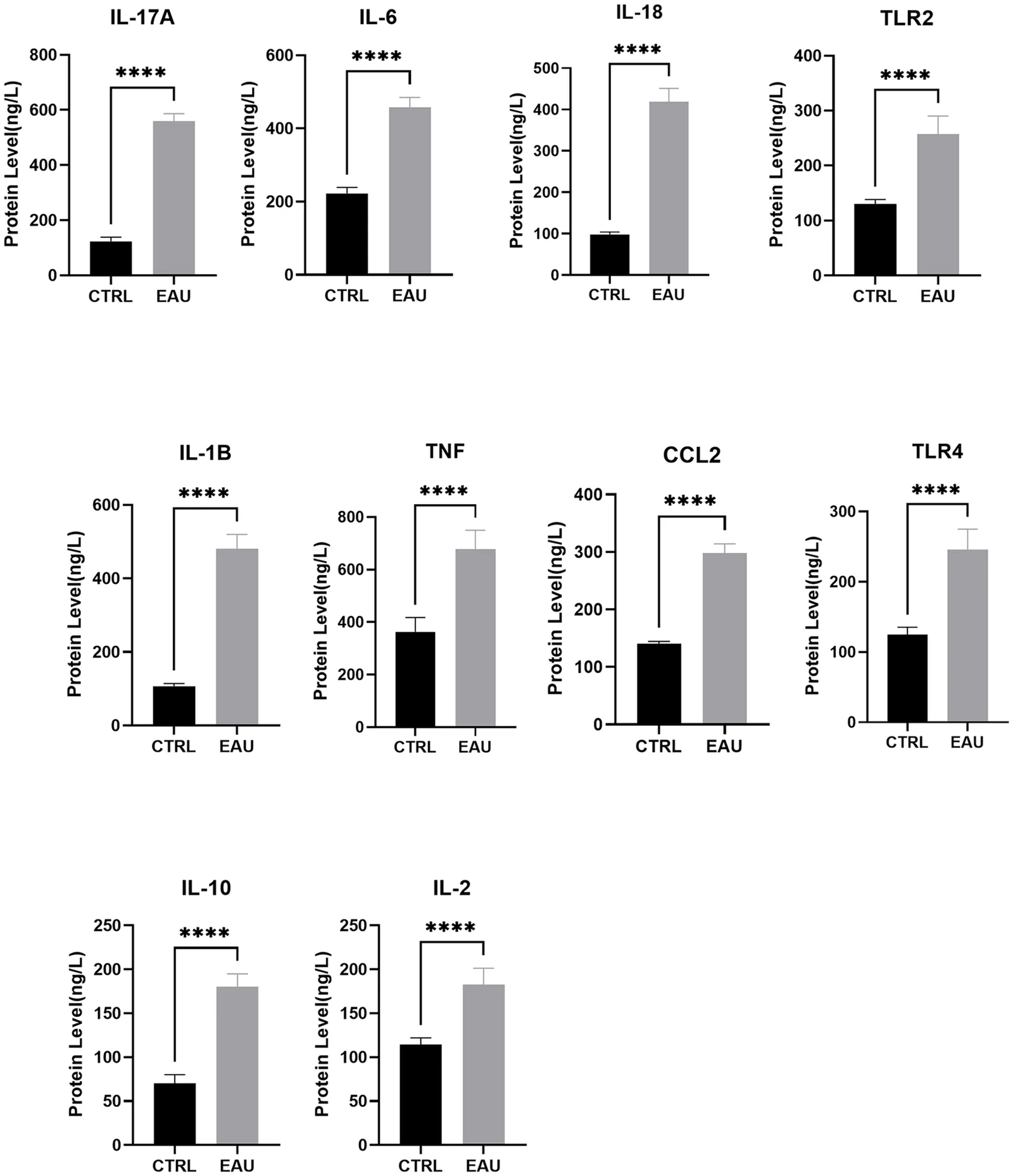
Protein expression levels of the hub genes in the liver and spleen tissues. Protein levels of IL-17A, IL-18, TLR2, IL-2, TLR4, IL-10, IL-1β, IL-6, TNF, and CCL2 in the liver and spleen of control and EAU rats 12 days after immunization. n = 10; ***p < 0.001.

### ELISA detection of inflammatory factors in the liver and spleen tissues of EAU rats.

ELISA results showed that the protein levels of IL-17A, IL-18, TLR2, IL-2, TLR4, IL-10, IL-1β, IL-6, TNF, and CCL2 in the liver and spleen tissues were significantly elevated in rats of the model group compared with the control group (p < 0.001); these findings were consistent with the trend of DEG mRNA expression in the EAU rat model (Fig 7).

## Discussion

Uveitis is a recurrent, non-limited ophthalmic diseaseaffecting the uvea, retina, and vitreous, which can lead to a variety of complications and can cause severe visual impairment, the prevalence of which is increasing year by year. There are more than 60 causes of uveitis. Previous reports suggest that 20–40% of uveitis is non-infectious. Some of these cases may be associated with systemic rheumatic and autoimmune diseases, but some affect only the eye. The epidemiology and clinical incidence of some forms of uveitis vary around the world due to genetic, ethnic, environmental and socio-economic factors [15]. Autoimmunity, autoinflammation and infection are important factors in the pathogenesis of uveitis, focusing on the role of innate and adaptive immune responses. Studies have shown that infection [16], either directly or indirectly, along with dysregulation of the microbiome, may contribute to the formation of immune-mediated ocular inflammation, and that the development of uveitis is a complex process involving the regulated expression of multiple genes, including immune and inflammatory factors. The aberrant expression of multiple factors plays an important role in the development of uveitis, which is difficult to prevent and has drawbacks in many treatment modalities in the clinic. Exploring its pathogenesis and finding new therapeutic targets has become an urgent issue in the treatment of uveitis.

Due to recent progress in protein chemistry, mass spectrometry and bioinformatics, bioinformatics has received increasing levels of attention in the field of uveitis research. In-depth analysis of autoimmune uveitis bioinformatics information can be used to identify valuable key genes and pathways for further analysis, thus revealing the pathological mechanisms underlying autoimmune uveitis and providing a theoretical basis for the targeted treatment of this disease. The pathogenesis of uveitis is complex and is influenced by the interactions between inflammation, immunity and genetics. Currently, potential therapeutic targets for many diseases can be predicted by microarray and high-throughput sequencing. In this study, data were extracted from the GSE66936_RAW dataset in the GEO database, and 129 DEGs were obtained between uveitis patients and healthy individuals; these DEGs are likely to have a close relationship with the pathogenesis of uveitis. In addition, 139 uveitis-related genes were obtained from six internationally recognized disease target gene databases (TTD, DrugBank, OMIM database, GAD, PharmGKB and DisGeNET databases). In order to better understand the functions of these DEGs, PPI network construction, GO analysis and KEGG analysis were further performed.

GO enrichment analysis of the above DEGs revealed that enrichment of the biological processes of uveitis-related genes predominantly involved cytokine activity, chemokine activity, leukocyte and granulocyte chemotaxis, inflammatory response involving monocyte migration, immune response mediated by B-cells, neutrophils, and lymphocytes, viral defense, cellular response to lipopolysaccharide, and the regulation of metabolism by molecules such as nitric oxide and neurotransmitters. Consequently, these molecules and other biological processes are involved in the pathogenesis of uveitis. Significant signaling pathways associated with the DEGs were mainly enriched in cytokine-cytokine receptor interaction, the TNF signaling pathway, the NF-κB signaling pathway, NOD-like receptor signaling pathway, Toll-like receptor signaling pathway, JAK-STAT signaling pathway, and the Cytosolic DNA-sensing pathway. A previous study [17] showed that autoimmune uveitis is considered to be an autoimmune disease mediated by CD4 + T cells, which mainly invade the retina and uvea. Primary CD4 + T cells are activated by T cell receptors and can differentiate into Th1, Th2, Th17 and Treg cells to participate in different types of immune responses. Antigen production by retinal and uveal cells activates the initial T cells, which undergo proliferation and differentiation and play a key role in inflammation by recruiting and activating monocytes, macrophages, T cells, and NK cells, thus enhancing their activity, as well as activating the Toll-like receptor signaling pathway, the NOD-like receptor signaling pathway, chemokine-mediated signaling pathway and other pathways, thus inducing the release of multiple cytokines, chemokines and adhesion molecules, mediating the adhesion of leukocytes to vascular endothelial cells. These events lead to the infiltration of inflammatory cells, thus resulting in an inflammatory response [18]. TLR signals act downstream through MyD88 to activate nuclear factor-κB (NF-κB), thus promoting the expression of NF-κB-regulated genes, which amplifies the inflammatory response cascade.

**It is noteworthy that the GSE66936 dataset originates from human CD14 and CD16 monocytes, which represent the innate immune compartment, whereas our experimental validation employed the EAU model focusing on CD4 + T cell-mediated adaptive immunity. This apparent disconnect is bridged by the well-established concept of innate-adaptive immune cross-talk in autoimmune uveitis [6]. Activated monocytes and macrophages serve as antigen-presenting cells (APCs) that process and present retinal antigens to naïve T cells in draining lymph nodes, initiating the adaptive immune response [7]. Monocyte-derived IL-6 and IL-1β provide essential cytokine signals that, together with TGF-β, drive the differentiation of naïve CD4 + T cells into pathogenic Th17 cells [19]. Similarly, monocyte-derived TNF-α and IL-12 promote Th1 cell polarization, which together with Th17 cells constitute the principal effector populations in EAU [20]. Furthermore, monocyte-secreted chemokines, including CCL2 and CCL5, create a chemotactic gradient that recruits activated T cells from the peripheral circulation into the intraocular space, perpetuating the inflammatory cascade [21]. TLR signaling in monocytes (notably TLR2 and TLR4, both identified as hub genes in our analysis) upregulates co-stimulatory molecules CD80 and CD86, which are essential for effective T cell priming and activation [22]. Thus, the monocyte transcriptomic profile captured by GSE66936 reflects the upstream innate immune events that subsequently orchestrate the adaptive T cell response validated in our EAU model.**

In addition, the results of GO enrichment analyses also included the metabolic regulation of cellular responses to lipopolysaccharide and molecules such as nitric oxide and neurotransmitters. A previous study [23] showed that lipopolysaccharide can induce the release of reactive oxygen species (ROS) from macrophages and neutrophils. ROS can also activate NF-κB to induce the release of cytokines, such as *IL-1* and *TNF-α*, to induce the generation of ROS. iNOS catalyzes the production of endogenous nitric oxide (NO), a cytotoxic effector molecule. NO can be released locally in inflammatory autoimmune diseases and cause tissue damage, thus exacerbating the degree of EAU pathogenesis. Furthermore, NO is also an important regulator of Th1/Th2 balance, which specifically down-regulates the Th1-type cytokines (IL-2 and IFN-γ),thus regulating the development of autoimmune uveitis and creating a vicious circle [24]. This may also be the key reason underlying refractory and recurrent autoimmune uveitis.

Many studies have shown that cytokines play an important role in the development of inflammation, and various inflammatory factors are known to be significantly elevated in the serum of patients with uveitis. Cytokines are low molecular weight and biologically active peptides that play important biological roles in cell-cell interactions, cell differentiation and activation, tissue repair and destruction [25]. Cytokines are released by a variety of cells, including lymphocytes, leukocytes, endothelial cells, macrophages, and natural killer cells, and are transmitted via hematogenous and/or endocrine pathways to affect other target cells in the periphery or at a distance. Certain interactions between pro-inflammatory and anti-inflammatory cytokines in tissues via hematogenous and/or endocrine pathways are essential for the maintenance of a healthy immune microenvironment. Previous studies have shown that dysregulation of the balance of anti-inflammatory and pro-inflammatory cytokines leads to a variety of autoimmune disorders such as systemic lupus erythematosus, rheumatoid arthritis, Bechet’s disease, inflammatory bowel disease, multiple sclerosis, immunosuppression, anti-allergic and immune deficiency responses, and tumor formation [26–29].

In order to search for key genes involved in the development of uveitis, we performed co-expression network analysis and protein interactions analysis on the obtained differential genes, and screened 10 key genes associated with the disease, including *IL 17A, IL-18, TLR2, IL-2, TLR4, IL-10, IL-1β, IL-6, TNF*, and *CCL2*. A previous study [30] found that Th17 dysregulation of regulatory T-cell balance plays an important role in the pathogenesis of autoimmunity. Th17 cells are pro-inflammatory cells that secrete a variety of cytokines, the most important of which are *IL 17* (also known as *IL 17A*), *IL 17F, TNF-α, IL-21*, and *IL-22*. Th17 cells were previously found to be up-regulated in a proportion of peripheral blood mononuclear cells in patients with active uveitis. The inhibition of their differentiation significantly attenuated the inflammatory response during the active phase of uveitis. Therefore, exploring the regulatory mechanisms of Th17 cells is crucial for the treatment of uveitis [31].

Previous studies have shown that inflammatory effector T cells (Th1 and Th17) play a crucial role in the development of EAU [32]. During the pathogenesis of EAU, the blood-ocular barrier is disrupted, and inflammatory effector T cells are recruited from peripheral lymphoid tissues to the retina, releasing inflammatory factors to destroy the tissue structure, thus creating a key pathological event.

TLRs are pattern recognition receptors in the body’s natural immune system and are transmembrane glycoproteins expressed by cells such as macrophages, neutrophils, epithelial cells, and endothelial cells, which play an important role in the body’s immune response to infections and cellular injuries. TLRs can be directly or indirectly involved in the development of a variety of diseases such as septicemia, chronic obstructive pulmonary disease (COPD), fungal infections,autoimmune diseases and cancer [33]. TLRs at different sites activate autoimmune effects by recognizing different types of associated molecular patterns, and when TLRs are activated by binding, they transmit signals into the cell and proceed to mediate several associated transcription factors such as *NF-κB*, activator protein-1 (AP-1), interferon regulatory factor 3 (IRF3), interferon regulatory factor 7 (IRF7) and other transcription factors. An important intermediary effector in this process is myeloid differentiation factor 88 (MyD88), which acts as an intracellular signal transduction hub and is responsible for collecting and activating various kinases and ubiquitin ligases, and then mediates downstream signal transduction. While numerous TLRs exist, toll-like receptor 2 (*TLR2*) is considered as the most important. Toll-like receptor 2 (*TLR2*) and *TLR4* are widely recognized PRRs at the cell membrane and are most effectively activated by ligands [18]. For example, stimulation of intestinal epithelial cells(IEC) cultured *in vitro* with LPS increases the expression of *TLR2* and *TLR4*. LPS can also be recognized by *TLR2* and *TLR4* receptors, which in turn activates the MyD88/NF-κB pathway and increases the expression of various inflammatory factors.

Many studies have reported high levels of interleukin-10 in patients with uveitis [34–36]. This increase in *IL-10* levels occurs in order to regulate activated pro-inflammatory pathways. Patients with active uveitis exhibited higher levels of *IL-10* than patients with inactive uveitis [37]. Similarly, El-Asrar et al. reported higher levels of *IL-10* in the AQH of patients with active uveitis than in normal human AQH and concluded that elevated levels of *IL-10* are a potential indicator of activation [38]. Furthermore, in addition to being associated with disease activation, findings have shown that *IL-10* is associated with infectious or non-infectious uveitis. Curnow et al. determined that there was no significant difference in *IL-10* levels when compared between patients with active idiopathic uveitis (n = 37) and normal controls (n = 12). Furthermore, Curnow et al. used polymerase chain reaction (PCR) to demonstrate that herpetic serum levels of IL-10 in patients with uveitis were significantly higher than in patients with non-infectious uveitis [39]. Takase et al. [25] found that *IL-10* levels in AQH of patients with infectious uveitis (n = 8) were significantly higher than those of patients with non-infectious uveitis (n = 9). Elevated levels of *IL-10* may be induced by infection, thus suggesting that *IL-10* plays the role of an activation marker.

TNF is a cytokine produced by activated macrophages, CD4 lymphocytes and natural killer cells. *TNF* mainly stimulates and maintains the inflammatory response in autoimmune diseases and plays an important role in maintaining the immune stability of the body. The abnormal expression of *TNF* can cause a variety of systemic or local diseases. In recent years, findings have confirmed that uveitis is mainly an autoimmune disease mediated by Th1 and Th17 cells [40]. In uveitis, TNF cytokines play an active role.Mizuki et al. [41] found that TNF-α levels in the peripheral blood increased dramatically during episodes of EAU and decreased in remission. The TNF signaling pathway can promote intraocular inflammation by regulating the immune-inflammatory response, inducing inflammatory cascade reactions and leading to dysfunction of the retinal pigment epithelial cells and affecting the functionality of the retinal barrier response [42]. *TNF* is one of the key cytokines in the occurrence and pathogenesis of uveitis and inhibiting the overexpression of *TNF* can be targeted to treat uveitis. Furthermore, anti-TNF biological agents can directly bind to the *TNF* receptor or *TNF*, inhibit the expression of *TNF*, and further inhibit macrophage activation, thus inhibiting the immune response and inflammatory process, thus representing an efficient treatment of uveitis [43–45].

The chemokine *CCL2* is an inflammatory cytokine produced by retinal cells that play a key role in inflammation by recruiting and activating monocytes, macrophages, T cells and NK cells. *CCL2* can respond to different inflammatory stimuli, including *TNF-α, IL-1β* and endotoxin [46]. As *CCL2* has an important role in vascular inflammation, its expression has been associated with a variety of acute and chronic inflammatory diseases. Elimination of the *CCL2* pathway by gene deletion or antibody neutralization has been shown to prevent the pathogenesis of many inflammatory diseases, including endotoxin-induced uveitis (EIU), retinal neovascularization and atherosclerosis. Thus, blocking *CCL2* production reduces the pathological signs of uveitis, ischemic retinopathy, and tissue destruction.

NF-κB is a nuclear transcription factor with multidirectional and multi-effect regulation, which can specifically bind to the κB sites of promoters or enhancers of various cytokines, adhesion molecules and other genes to promote the transcription and expression of these genes. Thus, *NF-κB* is closely related to the physiological and pathological processes of cell proliferation, differentiation, adhesion, apoptosis, inflammatory response and immune response, and plays an important role in growth, development, inflammation, immune response and tumor growth [47]. Studies have shown that *NF-κB* is closely related to the inflammatory response in uveitis, and its inhibition may be a new therapeutic target for uveitis [48]. Hsu et al. [49] investigated a model of EAU in which bortezomib, a proteasome inhibitor, was shown to inhibit the activation of NF-κB. This inhibited the expression of key inflammatory mediators, such as IL-1, IL-12, and IL-17, and also inhibited the expression of TNF-α, NF-κB, and MCP-1, thus ameliorating the ocular inflammation caused by autoimmune uveitis.Kubota et al. [42] used a model of endotoxic uveitis (EIU) to show that the expression of the inflammatory factors (ICAM-1 and MCP-1) was inhibited by the suppression of oxidative damage and the activation of NF-κB, thus inhibiting EIU in normal ocular structures.

Therefore, the present study focuses on the activation of the inflammatory response in autoimmune uveitis, and identifies key DEGs (*IL 17A, IL-18, TLR2, IL-2, TLR4, IL-10, IL-1β, IL-6, TNF,* and *CCL2*) in an animal model. The rat model of EAU is a well-recognized and widely-used animal model of autoimmune uveitis, and the pathogenesis and clinical manifestations of EAU are extremely similar to those of human uveitis and can simulate changes in the organism in the disease state. In the present study, we generated a rat model of EAUby subcutaneously injecting interphotoreceptor retinoid-binding protein (IRBP) antigen; then, we scored the ocular tissues of the rats using a clinical score. Observation of pathogenic symptoms and pathological tests showed that rats in the model group showed a range of clinical symptoms, such as iris vascular congestion, moderately opaque dark red reflexes in the anterior chamber, and pupillary constriction under slit lamp microscopy. Furthermore, pathological changes such as retinal structural disorders, edema, and infiltration of inflammatory cells, were evident on HE staining, along with elevated clinical scores, thus indicating that the animal model of EAU had been successfully established, the results were stable, and that it would be useful for investigating the causative agents of autoimmune uveitis in humans. This model laid the foundation for investigating the pathogenic mechanisms and treatment strategy of human autoimmune uveitis, and can provide the next direction and foundation for mechanistic studies. In this study, the expression of *IL 17A, IL-18, TLR2, IL-2, TLR4, IL-10, IL-1β, IL-6, TNF* and *CCL2* genes and proteins in the ocular tissues and aqueous humor of rats in the model group were significantly elevated; these changes may play key roles in the pathogenesis of uveitis [40],consistent with the results previously published by Ongkosuwito et al. [34].

After the EAU model had been successfully induced, the levels of cytokines in the EAU model were detected by ELISA and then compared with the control group. The most significant increase in the expression of *IL 17* was observed in the EAU model group, thus indicating that *IL 17* was involved in the process of EAU and might play a key role in the initiation and maintenance of the disease, thus providing a basis for the involvement of Th17 cells in the induction of autoimmunity. The increased expression of the *IL-18* gene suggested that *IL-18* may promote the production of *IFN-γ* by enhancing specific cell-mediated immune responses, thus enhancing the immune response of Th1 cells, amplifying the inflammatory response, and exacerbating inflammation occurring in the tissues to cause severe damage. In the present study, we showed that *IL-6* is rapidly elevated in inflammation and is able to promote and activate T-cells, stimulate B-cell differentiation, stimulate immunoglobulin secretion, promote acute phase protein synthesis and platelet production, and induce the production of other growth factors that are involved in the inflammatory response. The release of these factors further promotes platelet activation, aggregation, and secretory effects leading to a cascade of amplifying effects. The simultaneous recruitment of a large number of inflammatory cells, such as lymphocytes and macrophages, and the promotion of their activation and secretion, may suggest that elevated levels of *IL-6* may be associated with the intensification of inflammatory responses and tissue damage. During the process of Th17 cell production and differentiation, IL-6 and TGF-β work together to induce the differentiation of T cells into Th17 cells. In addition, the induced Th17 cells secrete *IL-6*, thus creating positive feedback between the two molecules. *IL-2* is mainly produced by activated T cells and plays a key role in enhancing T cell proliferation and activity. Furthermore, IL-2 plays a crucial role in regulating the balance of the immune system, especially in maintaining the function of Treg cells. Alterations in the gene and protein expression of *IL-2* lead to an imbalance of *IL-2* which may lead to dysregulation of the inflammatory response in the uvea. Changes in mRNAs related to key signaling pathways were detected by real-time PCR; resultant trends were the same as those of ELISA. While the anti-inflammatory factor *IL-10* can significantly reduce the production of inflammatory mediators, such as inhibiting the secretion of cytokines (*TNF-α, IL-1*, and *IL-6*), it can also reduce the activation and infiltration of inflammatory cells and inhibit the response of Th1 and Th17 cells. Most importantly, IL-10 enhances the functionality of regulatory T-cells (Treg cells), inhibits the activation of dendritic cells and macrophages, and reduces their activating effect on T cells, which is essential for maintaining balance in the immune system and preventing excessive immune responses. By counteracting EAU by reducing inflammation and immune responses, *IL-10* may help to protect ocular tissues from excessive immune responses and inflammatory damage. Th1 and Th17 jointly mediate the pathogenic process of EAU. It is also evident that Th1 and Th17 can cross-regulate each other. Th17 cells alone cannot induce autoimmune diseases; the participation of Th1 is also necessary. However, the specific interactions between these two molecules in the pathogenesis of autoimmune diseases still need to be investigated. Th1 and Th17 cells, together with Th2 and Treg cells, create a three-dimensional cytokine regulatory network to maintain the body’s immune homeostasis and regulate balance in the cytokine network, thus providing an effective way to prevent and treat uveitis. The relationship between *TLR2/4* and autoimmune uveitis has been reported in several papers. TLR represents a bridge between natural and acquired immunity. The endotoxin activation of *TLR4* induces a standard model of uveitis in rats, as verified by our current experiments.

Furthermore, our findings suggest that the cytokine microenvironment in EAU overrides multiple physiological checkpoints that normally maintain immune tolerance. First, sustained IL-6 and IL-1β signaling, originating from activated monocytes and macrophages, overrides the Treg-mediated suppression checkpoint by inhibiting FOXP3 expression and promoting Th17 differentiation [50]. This creates a self-amplifying loop where Th17 cells produce additional IL-6 and IL-17, further destabilizing the Treg/Teff balance. Second, TNF-α-mediated NF-κB activation upregulates PD-L1 expression on antigen-presenting cells, which, paradoxically, can create a feedback loop that dysregulates T cell activation thresholds and promotes chronic inflammation [51]. Third, the chemokine gradient established by CCL2 and CCL5 overrides the blood-retinal barrier checkpoint by promoting leukocyte transendothelial migration and intraocular infiltration [52]. The multi-layered checkpoint disruption revealed by our integrated bioinformatics and experimental approach underscores the complexity of autoimmune uveitis and highlights the need for therapeutic strategies that target multiple nodes simultaneously.

Several limitations of this study should be acknowledged. First, pathway activation in this study was inferred from transcriptomic enrichment analyses together with endpoint measurements of cytokine mRNA and protein levels by RT-PCR and ELISA; direct protein-level evidence, such as Western blot analysis of phosphorylated signaling proteins, and in situ validation, such as immunohistochemistry or flow cytometric analysis of T cell subsets, were not performed and will be important directions for our future work. Second, the GSE66936 dataset is derived from human monocytes (innate immunity), whereas the EAU validation primarily reflects T cell-mediated adaptive immune responses; the innate-adaptive immune cross-talk discussed above should therefore be interpreted as a literature-supported hypothesis rather than as a mechanism directly demonstrated by our data. Nevertheless, the concordance between the bioinformatic predictions and the experimental cytokine data supports the reliability of the hub genes identified in this study.

## Conclusions

In this study, 139 genes related to the pathogenesis of uveitis were obtained by GEO analysis of the GSE66936 dataset and by searching internationally recognized disease gene-related databases. We also constructed interaction networks for the 139 targets and performed GO and KEGG analysis on the targets. Analysis showed that the pathogenesis of autoimmune uveitis was associated with the differential expression of a range of key genes in ocular tissues, and that the inflammatory and immune responses of uveitis mainly involved cytokine-cytokine receptor interactions, the TNF signaling pathway and the NF-κB signaling pathway, thus suggesting that the pathological process of autoimmune uveitis might involve the activation of T-cells by the retinal antigens *in vivo.* These cells then undergo proliferation and the activated retinal antigen-specific T cells migrate from the draining lymph nodes and/or spleen into the eye, recognize intraocular antigens, destroy the blood-retinal barrier, and through the secretion of adhesion molecules and chemokines, further recruit T cells, monocytes, macrophages, and neutrophils to enter into the eye, amplify the inflammatory response, and lead to parenchymal cell damage. The findings of the present study provide new ideas to unravel the pathogenesis of uveitis and provide targets for the effective treatment of uveitis. Follow-up experiments can explore these proteins and pathways to study the specific mechanisms of uveitis and identify new biomarkers, thus providing a new direction for the targeted treatment of uveitis.

## Supporting information

S1 Data DEG list from GEO. (XLSX)

S2 Data Disease related genes. (XLSX)

S3 Data GO enrichment. (XLSX)

S4 Data KEGG pathway enrichment. (XLSX)

S5 Data PPI network genes. (XLSX)

S6 Data Hub gene ROC. (XLSX)

S7 Data RT-PCR Ct fold change. (XLSX)
